# Green and mild retro-aldol synthesis of natural benzaldehyde from cinnamaldehyde over Li-doped MgO catalysts

**DOI:** 10.1039/d5ra07239e

**Published:** 2025-11-17

**Authors:** Ngoc Quang Phan, Duc Chinh Pham, Thi Uyen Nguyen, Dang Van Do, Thu Thi Minh Nguyen, Hong Duc Ta

**Affiliations:** a School of Chemistry and Life Science, Hanoi University of Science and Technology No. 1 Dai Co Viet Hanoi 100000 Vietnam duc.tahong@hust.edu.vn; b Faculty of Chemistry, VNU University of Science No. 19 Le Thanh Tong Hanoi Vietnam

## Abstract

A green and efficient method for the selective synthesis of natural benzaldehyde (BA) from cinnamaldehyde (CA) was developed using Li-doped MgO (Li/MgO) catalysts under mild conditions. Catalysts were systematically characterized *via* SEM, XRD, CO_2_-TPD, and NH_3_-TPD to investigate their structural and surface properties. Reaction parameters, including solvent composition, water content, catalyst dosage, reaction temperature, and stirring speed, were optimized. Among the tested materials, 0.25 Li/MgO delivered the highest benzaldehyde yield of 40.65% within 3 hours under optimal conditions: 0.01 mole CA, CA/H_2_O molar ratio of 1 : 83, CA/EtOH ratio of 1 : 25, catalyst loading of 0.006 g mL^−1^, 343 K, and 600 rpm. Catalyst reusability tests demonstrated stable performance over four cycles without significant loss of activity. Kinetic analysis followed a pseudo-first-order model, yielding a rate constant of 0.0032 min^−1^ and an activation energy of 22.73 kJ mol^−1^. A retro-aldol reaction mechanism involving active surface sites on the catalyst was proposed. Importantly, scale-up experiments using 10-fold reactant quantities maintained comparable yields, underscoring the catalyst's scalability and industrial relevance. This work presents a sustainable and selective approach for natural benzaldehyde production, with both the catalytic system and mild operational conditions offering significant advantages in green chemistry.

## Abstract 2

A green and efficient method for the selective synthesis of natural benzaldehyde (BA) from cinnamaldehyde (CA) was developed using Li-doped MgO (Li/MgO) catalysts under mild conditions.

## Graphical abstract

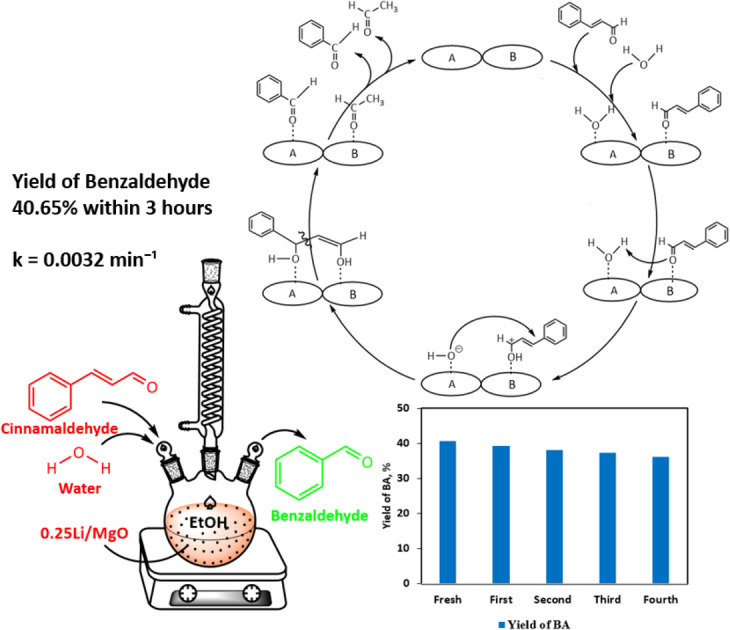

## 1. Introduction

In recent years, the increasing demand for natural product-based ingredients has been driven by consumer concerns over food safety and sustainability. Among natural aroma compounds, benzaldehyde (BA) is the second most widely used flavoring agent after vanillin, with extensive applications across the food, fragrance, and pharmaceutical industries.^1^ However, the production of natural BA remains limited, resulting in significantly higher costs compared to synthetic alternatives.^2–4^

Traditionally, natural BA is produced *via* the alkaline hydrolysis of amygdalin (laetrile), a process that generates toxic hydrogen cyanide (HCN) as a byproduct, raising environmental and safety concerns and violating the principles of green chemistry. As an alternative, cinnamaldehyde (CA), the primary component (>75%) of cinnamon cassia oil (CCO),^5^ has emerged as a promising, sustainable precursor for natural BA synthesis through retro-aldol conversion.^6^ This transformation is particularly significant because it enables the valorization of renewable, biomass-derived cinnamaldehyde into benzaldehyde without generating hazardous cyanogenic by-products.^7–9^ Nevertheless, this route is *indirect* and thermodynamically less favorable, as cinnamaldehyde itself can be synthesized from benzaldehyde *via* aldol-condensation with acetaldehyde.^10,11^ Therefore, the retro-aldol conversion of CA to BA represents a reversible process often constrained by limited equilibrium conversion and selectivity, underscoring the importance of catalyst design and reaction optimization.^7,12^ Despite these intrinsic limitations, this pathway remains an attractive green alternative for producing natural-grade benzaldehyde when combined with efficient, recyclable heterogeneous catalysts under mild conditions.

On the other hand, CA's poor water solubility and reactivity under neutral conditions limit the reaction yield. To address this, various methods have been explored, including: hydrolysis in near-critical wast,^13^ hydrotalcite-based solid base catalysis,^14^ amino acid ionic liquids,^15^ and phase-transfer catalysis using β-cyclodextrin or its derivatives.^16,17^ Other approaches include oxidative cleavage using heterogeneous cyclodextrin-based catalysts or Zr-modified ZnO systems,^18–23^ as well as reactive distillation integrated with alkaline hydrolysis.^1^ While many of these techniques provide moderate to high yields, they suffer from critical drawbacks such as harsh conditions, expensive or non-recyclable catalysts, catalyst recovery challenges, or complex reactor setups, thereby limiting their industrial viability.

For example, hydrolysis in near-critical water can improve solubility but requires hard reaction conditions (553 K, 15 Mpa), compared to the natural quality of the BA.^13^ Solid base hydrotalcites offer improved yield (∼70%) but are difficult to scale due to intricate equipment design.^14^ Ionic liquids, although effective, are costly and difficult to separate.^15^ Cyclodextrin-assisted methods yield well but often require lengthy reaction times and involve complex or non-recyclable catalysts.^16–19^ Heterogeneous cyclodextrin-functionalized catalysts improve recyclability but involve labor-intensive synthesis and sometimes require strong oxidants, which may degrade the product aroma profile. Therefore, there is a clear need for a mild, green, and economically scalable route for converting CA to BA, using environmentally benign and easily recoverable heterogeneous catalysts. Among potential candidates, magnesium oxide (MgO) is a cost-effective, non-toxic, and thermally stable solid base with favorable surface basicity.^24–26^ MgO has been widely applied in various catalytic transformations such as aldol condensation, transesterification, CO_2_ utilization, and biomass conversion owing to its tunable surface chemistry and structural robustness. Recent studies have further demonstrated the potential of MgO-based systems for sustainable catalysis. For instance, Li *et al.* developed supported MgO catalysts for the green synthesis of diethyl carbonate from ethyl carbamate and ethanol, while Man *et al.* reported Zn/MgO nanoplates for biodiesel production from *Camelina* oil with high activity and recyclability, and Jiang *et al.* showed that K/Ni–MoS_2_/MgO catalysts facilitate CO_2_ hydrogenation to methanol under mild conditions.^27–29^ Other reports highlight MgO's synergy in multicomponent systems, such as Ni/MgO–MO_*x*_ (M = Zr, Ti, Al) and Fe_2_O_3_–MgO composites, further emphasizing its versatility in green catalysis.^30,31^ Collectively, these findings reaffirm MgO's status as a robust and recyclable heterogeneous catalyst with great potential for environmentally friendly chemical transformations.

Despite these advances, the application of MgO to retro-aldol reactions**,** particularly the cleavage of CA to BA, remains largely unexplored. Moreover, alkali-metal doping (*e.g.*, Li, Na, K) can significantly enhance the surface basicity and catalytic activity of MgO, presenting a promising route for improving reactivity and selectivity. Compared with previously reported homogeneous and heterogeneous systems, the Li/MgO catalyst developed in this work uniquely combines enhanced basicity, low activation energy, and excellent reusability for the retro-aldol cleavage of cinnamaldehyde, providing a novel, green catalytic route to natural benzaldehyde.

**Scheme 1:**
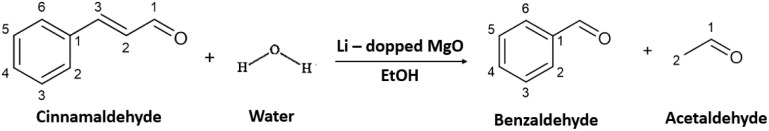
Green synthesis of BA from CA catalyzed by Li-doped MgO.

In this study, a series of alkali-doped MgO catalysts (A/MgO, where A = Li, Na, K) were synthesized and evaluated for their ability to catalyze the retro-aldol transformation of CA to BA under mild aqueous-ethanol conditions. The catalysts were thoroughly characterized using SEM, XRD, CO_2_-TPD, NH_3_-TPD, and surface area analysis. The study also aimed to (i) optimize the reaction conditions for maximum BA yield, (ii) assess catalyst reusability, (iii) conduct kinetic studies to determine activation energy and rate constants, and (iv) propose a plausible reaction mechanism. This work provides a green, scalable, and economically viable approach for producing natural BA using heterogeneous catalysis under environmentally friendly conditions. To the best of our knowledge, this is the first report demonstrating the use of Li-doped MgO as a heterogeneous catalyst for the selective retro-aldol conversion of cinnamaldehyde to benzaldehyde under mild, environmentally friendly conditions (Scheme 1).

## 2. Experimental section

### 2.1. Chemicals

Cinnamaldehyde (>98%) was purchased from Tokyo Chemical Industry Co., Ltd, Tokyo, Japan. Absolute ethanol (99.8%) was obtained from Fisher Chemical, Geel, Belgium. Magnesium hydroxide (Mg(OH)_2_, 95%) and lithium hydroxide monohydrate (LiOH·H_2_O, 98.5%) were supplied by Sigma-Aldrich. Sodium hydroxide (NaOH, 97%) was purchased from VWR Chemicals BDH, and potassium hydroxide (KOH, 99%) from Thermo Fisher Scientific. Ultrapure water was used throughout all experiments. All reagents were used as received without further purification.

### 2.2. Catalyst preparation

#### 2.2.1. Preparation of MgO

Magnesium oxide (MgO) was synthesized *via* thermal decomposition of commercial magnesium (Mg(OH)_2_). The precursor was subjected to a two-step calcination process in the air: initially heated at a rate of 1.1 K min^−1^ to 393 K and held for 1 hour, followed by a continued increase to 813 K at the same ramp rate and maintained for 12 hours to complete the conversion to MgO.

#### 2.2.2. Synthesis of alkali-doped MgO (A/MgO)

Alkali-doped MgO catalysts (A/MgO, where A = Li, Na, or K) were prepared using a conventional wet impregnation method. Briefly, the corresponding alkali hydroxide was dissolved in 50 mL of ultrapure water to form a homogeneous aqueous solution. MgO powder was then added to achieve a final molar ratio of A/Mg = 0.10. The resulting slurry was concentrated by rotary evaporation under vacuum. The obtained solid was subsequently calcined under the same conditions as described for pristine MgO. The final catalysts were designated as A/MgO.

#### 2.2.3. Optimization of Li doping level

After identifying lithium as the most effective dopant, a series of Li-doped MgO catalysts was synthesized with varying Li/Mg molar ratios (0.10, 0.15, 0.20, 0.25, and 0.30) to optimize the dopant loading. The materials were prepared using the same wet impregnation and calcination protocol described above. The resulting catalysts were designated as *x*Li/MgO, where *x* corresponds to the respective molar ratio.

### 2.3. Catalyst characterizations

The physicochemical properties of the catalysts were thoroughly characterized using multiple analytical techniques. Specific surface areas were determined *via* nitrogen adsorption–desorption isotherms at 77 K using a Horiba Scientific SA-9600 Series instrument after degassing the sample under vacuum at 523 K for 8 h. Surface morphology was examined by scanning electron microscopy (SEM, Hitachi SU8020, Germany) operated at 2 kV. Crystalline phases were identified by X-ray diffraction (XRD) using a STOE STADI-P diffractometer equipped with a Dectris Mythen 1 K detector and Cu-Kα_1_ radiation (*λ* = 0.154 nm) under 40 kV and 40 mA; patterns were collected in the 2*θ* range of 3.00°–96.45°. Surface basicity was probed using CO_2_ temperature-programmed desorption (CO_2_-TPD) performed on a Micromeritics AutoChem II 2920 system with a thermal conductivity detector (TCD). Acidity was similarly assessed using NH_3_-TPD, with desorption conducted up to 1073 K. Quantitative analysis of trace elements was conducted using inductively coupled plasma mass spectrometry (ICP-MS) on an Elan 9000 (PerkinElmer). The quantitative analysis of benzaldehyde was conducted *via* proton nuclear magnetic resonance (^1^H NMR) spectroscopy using a Bruker AVANCE III HD Nanobay 400 MHz spectrometer in C_6_D_6_; chemical shifts (*δ*) were reported in ppm, and spectra were processed using TopSpin 3.2.

### 2.4. Reaction procedure

All reactions were conducted in a 100 mL three-necked round-bottom flask equipped with a reflux condenser and a magnetic stirring bar, immersed in a thermostatically controlled oil bath. In each experiment, 0.18 g of A/MgO catalyst (equivalent to 0.006 g mL^−1^), ultrapure water (H_2_O) with an initial cinnamaldehyde-to-water (CA/H_2_O) molar ratio of 1 : 83, and ethanol (EtOH) with a cinnamaldehyde-to-ethanol (CA/EtOH) molar ratio of 1 : 25 were introduced into the reactor and heated to the desired reaction temperature. Subsequently, 1.35 g of CA (0.01 mole) was rapidly added under continuous stirring. The reaction mixture was stirred for 30 minutes before sampling. Reaction progress was monitored by withdrawing 0.5 mL aliquots at 30 minute intervals using a glass pipette. Each sample was transferred into a 2 mL vial and stored in a refrigerator for 20 minutes to quench the reaction. Solid catalysts were separated by centrifugation at 6000 rpm for 3 minutes at room temperature (MIKRO 12-24). The benzaldehyde yield was quantitatively determined using proton nuclear magnetic resonance (^1^H NMR) spectroscopy (Bruker AVANCE III HD Nanobay 400 MHz Ultrashield) with benzene-d_6_ (C_6_D_6_) capillary as the internal standard. All experiments were repeated three times. The benzaldehyde yield (%) was calculated using the following equation:
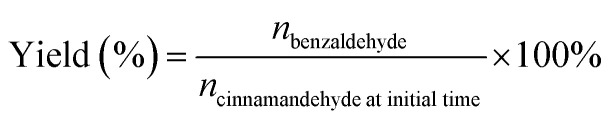


#### 2.4.1. Benzaldehyde

^1^H NMR (400 MHz, C_6_D_6_): *δ* 10.07 (s, 1H), 8.03 (d, 2H), 7.83 (t, 1H), 7.7 (t, 2H).

#### 2.4.2. Acetaldehyde

^1^H NMR (400 MHz, C_6_D_6_): *δ* 1.57 (d, 3H), 5.4 (q, 1H).

### 2.5. Kinetic investigations

A mixture of 0.18 g (0.006 g mL^−1^) 0.25 Li/MgO, 15 mL ultrapure water, and 15 mL EtOH was introduced into a 100 mL three-necked flask. The reaction temperature was controlled at 313 K, 323 K, 333 K, and 343 K. Then, 1.35 g (0.01 mol) of CA was added rapidly to the solution while being stirred for 30 minutes. The reaction mixture of 0.5 mL was sampled at 30 minute time intervals and then transferred into a 2 mL vial to store in the fridge for 20 minutes to quench the reaction. After that, the solution was centrifuged at 6000 rpm for 3 minutes at room temperature (MIKRO 12-24) to remove the solid catalysts in the samples. All experiments were repeated three times. The benzaldehyde yield was calculated *via*^1^H-NMR spectra with C_6_D_6_ capillary.

### 2.6. Scale-up experiment

The scale-up experiments were carried out under the optimum reaction conditions as follows: 150 mL EtOH, 150 mL ultrapure water, and 1.8 g 0.25 Li/MgO were placed in a 500 mL three-necked flask. The mixture was heated to 343 K in an oil bath, and then 13.5 g cinnamaldehyde was rapidly dropped into the mixture while being stirred for 30 minutes. After the reaction time, 0.5 mL of the sample was taken from the reactor by a glass pipette and then moved into a 2 mL plastic vial to be stored in the fridge to dampen the response. Subsequently, the sample was centrifuged at rpm for 3 minutes at room temperature (MIKRO 12-24) to remove the solid catalysts in the samples. The benzaldehyde yield was calculated through ^1^H-NMR spectra with C_6_D_6_ capillary.

## 3. Results and discussion

### 3.1. Characterization

**Fig. 1:**
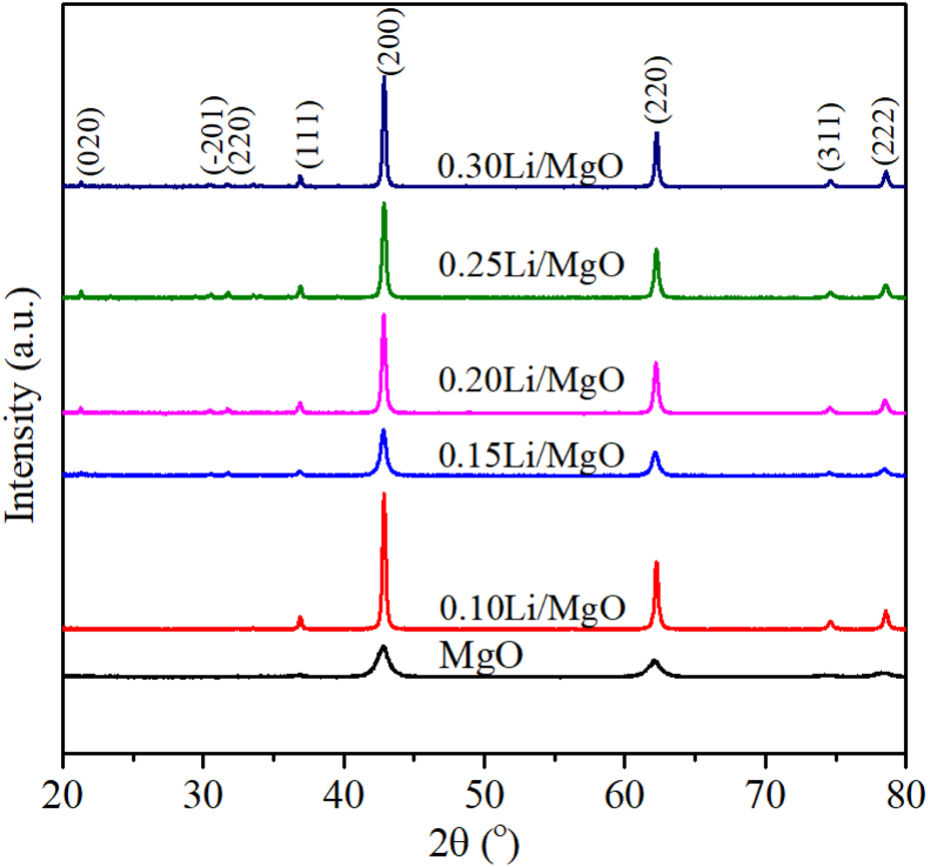
X-ray diffraction patterns of MgO and *x*Li/MgO.

Fig. 1 presents the XRD patterns of pristine MgO and *x*Li/MgO catalysts. The diffraction peaks of MgO are indexed to the (111), (200), (220), (311), and (222) planes, consistent with prior studies.^32^ The average crystallite sizes, estimated from the (200) reflection using the Scherrer equation, increased from 8.38 nm for pristine MgO to 31.53 nm for 0.30 Li/MgO. This growth trend indicates that Li incorporation promotes particle coalescence and partial crystallite aggregation, leading to a reduction in surface area and increased structural ordering. In addition to the MgO reflections, new diffraction peaks appearing at (020), (201), and (220) are assigned to LiOH·H_2_O, consistent with earlier studies.^33^ The presence of this hydrated phase arises from the rapid hydration of Li_2_O—formed *via* LiOH decomposition—upon exposure to ambient moisture at room temperature. The intensity of the LiOH·H_2_O peaks becomes more pronounced with increasing Li content, suggesting progressive formation of this segregated phase. The accumulation of Li-containing hydroxides on the surface may modify the catalyst's physicochemical characteristics by influencing surface basicity and partially blocking MgO active sites, thereby affecting catalytic performance.

Overall, the coexistence of MgO and LiOH·H_2_O phases points to incomplete incorporation of Li into the MgO lattice, highlighting the importance of optimizing Li content to balance structural integrity and surface functionality.

**Fig. 2:**
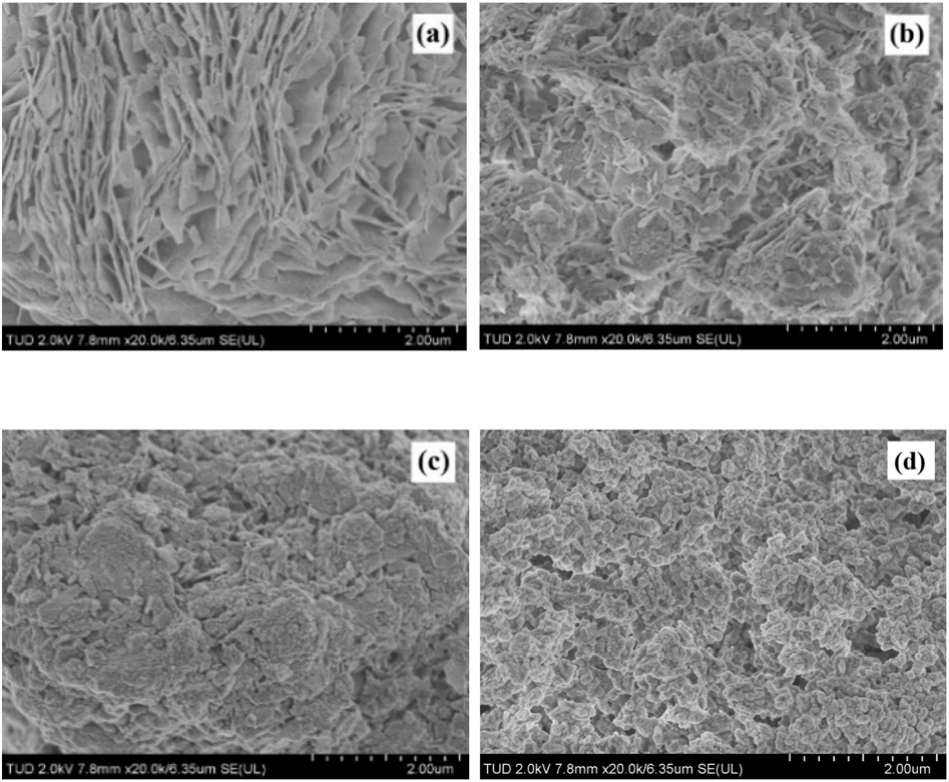
SEM images of (a) MgO, (b) 0.15 Li/MgO, (c) 0.25 Li/MgO, (d) 0.30 Li/MgO.

The surface area trends align closely with SEM observations. As shown in Fig. 2a, MgO synthesized from Mg(OH)_2_ exhibits a distinct flake-like morphology, which typically provides a high surface area beneficial for catalytic reactions. In contrast, *x*Li/MgO samples display progressively flatter and smoother particles with increasing Li content, as evident in Fig. 2b–d for 0.15 Li/MgO, 0.25 Li/MgO, and 0.30 Li/MgO, respectively. This morphological transformation correlates with a noticeable decrease in surface area, which can be attributed to particle agglomeration induced by the presence of Li salts during the aqueous impregnation step. Additionally, during the thermal decomposition of the LiOH/Mg(OH)_2_ precursor, the melting of LiOH likely promotes particle coalescence, further reducing porosity and surface roughness. These changes in surface texture and surface area are critical, as they may influence the availability and accessibility of active sites, potentially affecting catalytic activity. While particle agglomeration generally reduces surface area, the smoother morphology might enhance certain surface interactions or stability under reaction conditions, suggesting a trade-off between surface area and catalyst robustness that warrants further investigation.

**Fig. 3:**
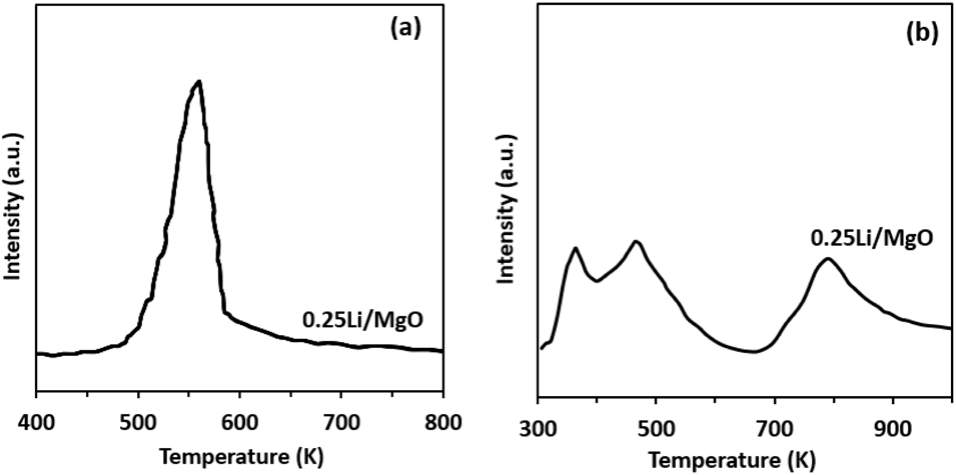
CO_2_-TPD spectra (a) NH_3_-TPD spectra (b) of 0.25 Li/MgO. An increase in the Li/Mg molar ratio resulted in a pronounced decrease in the surface area.

The surface basicity and acidity of the 0.25 Li/MgO catalyst were assessed using CO_2_-TPD and NH_3_-TPD, as presented in Fig. 3. The CO_2_-TPD profile in Fig. 3a exhibits a single desorption peak, indicating uniform basic sites on the catalyst surface, which are primarily associated with surface O^2−^ species. Such basic sites are known to facilitate reactions involving acidic intermediates or CO_2_ adsorption. In contrast, the NH_3_-TPD profile in Fig. 3b reveals three distinct desorption peaks, corresponding to weak, medium, and strong acidic sites. The coexistence of both basic and acidic sites suggests that 0.25 Li/MgO possesses biofunctional surface properties, which could enhance catalytic performance in reactions requiring both acid- and base-catalyzed steps. Moreover, the distribution and strength of these sites likely result from Li incorporation, which modifies the electronic environment and surface structure of MgO.

Quantitative integration of the CO_2_^−^ and NH_3_-TPD desorption peaks was performed to determine the total concentrations of basic and acidic sites (Table S2, SI). The total basic site concentration increased from 0.42 mmol g^−1^ for pristine MgO to 0.57 mmol g^−1^ for 0.25 Li/MgO, while the total acidity of 0.25 Li/MgO was 0.21 mmol g^−1^. These comparable magnitudes of acid and base site densities confirm the presence of well-balanced bifunctional active sites, consistent with the mechanism proposed for the retro-aldol conversion of CA to BA.

As summarized in Table 1, the surface area decreased from 130 m^2^ g^−1^ for pristine MgO to 88, 23, 15, and 9 m^2^ g^−1^ for Li/MgO samples with progressively higher Li loadings. This significant reduction can be attributed to particle agglomeration and pore blockage induced by Li salt impregnation, as well as the melting of LiOH during the thermal decomposition of the LiOH/Mg(OH)_2_ precursor. Such morphological changes, observed in the SEM micrographs, reduce the external surface available for adsorption and catalysis. While a lower surface area generally implies fewer accessible active sites, the concurrent changes in surface chemistry introduced by Li species may enhance catalytic performance in reactions that benefit from increased basicity, potentially compensating for the loss in surface area.

**Table 1:** Composition and catalytic activity of A/MgO catalysts^a^

Catalyst	Molar ratio (A/Mg)	Yield of BA (%)
Without catalyst	—	0.22
MgO	—	2.31
Li/MgO	0.10	32.67
Na/MgO	0.10	2.52
K/MgO	0.10	20.48

a Reaction conditions: CA (0.01 mole), CA/EtOH molar ratio (1 : 25), CA/H_2_O molar ratio (1 : 83), A/MgO (0.006 g mL^−1^), 343 K, 3 h, and 600 rpm.

### 3.2. Catalytic performance in retro-aldol cleavage

#### 3.2.1. Selection of the alkaline metal doping

The catalytic activity of the synthesized materials was examined in the retro-aldol cleavage of CA to natural BA, as shown in Table 1. In the absence of a catalyst, the benzaldehyde yield was negligible at 0.22%, confirming that the reaction does not proceed at an appreciable rate without a catalyst. Pristine MgO increased the yield to 2.31%, demonstrating that the surface basic sites on MgO can initiate the retro-aldol pathway but are insufficient in number or strength to achieve high conversion under the present conditions.

Alkali metal doping significantly altered the catalytic behavior. Li/MgO and K/MgO achieved yields of 32.67% and 20.48%, respectively, after 3 h, indicating that both dopants enhance the number or accessibility of catalytically active sites. This improvement is consistent with the hypothesis that alkali dopants introduce or strengthen surface basic sites (*via* modification of Mg–O bond character) and can also create a more favorable acid–base balance for retro-aldol cleavage. The superior performance of Li/MgO over K/MgO may stem from Li^+^'s smaller ionic radius, which allows for stronger interaction with the MgO lattice and more homogeneous distribution of active species. Li doping also likely promotes the coexistence of medium-strength basic sites, as observed in the TPD results, enabling a synergistic activation of the carbonyl group and β-C–H bond in CA.

In contrast, Na/MgO exhibited only a slight yield increase (2.52%), comparable to pristine MgO, suggesting that Na doping does not significantly alter the surface basicity or acid–base site distribution under these preparation conditions. This could be due to less effective dispersion of Na species or the formation of inactive sodium phases that block active MgO sites.

These findings clearly indicate that Li doping not only modifies the electronic structure and surface chemistry of MgO but also optimizes the acid–base site balance critical for retro-aldol reactions. While higher Li loading can reduce surface area due to particle agglomeration, the resulting enhancement in site functionality outweighs the geometric losses, leading to improved catalytic performance. Therefore, Li was selected for further doping studies to determine the optimal Li/Mg ratio for maximum benzaldehyde yield.

#### 3.2.2. Chemical and structural properties of *x*Li/MgO catalysts

Li was doped onto MgO with Li/Mg molar ratios ranging from 0.1 to 0.30, as confirmed by elemental analysis, and the corresponding chemical composition, surface area, and catalytic performance are summarized in Table 2. Increasing Li loading resulted in a systematic decrease in surface area, consistent with the SEM-observed morphological changes and attributed to particle agglomeration and pore collapse during preparation. Despite this reduction, catalytic activity in the retro-aldol cleavage of cinnamaldehyde did not decrease proportionally. 0.10 Li/MgO achieved a benzaldehyde yield of 32.67%, higher than undoped MgO, while 0.25 Li/MgO exhibited the highest yield of 40.65%, followed closely by 0.30 Li/MgO at 38.10%. These results indicate that moderate-to-high Li loadings enhance the density and strength of bifunctional acid–base sites sufficiently to offset the loss of surface area. At low loading, the basic site enhancement is modest, yielding only incremental improvements; at intermediate loading (0.25 Li/MgO), the balance between acid–base site distribution and accessibility appears optimal for simultaneous activation of the carbonyl group and β-C–H bond, maximizing catalytic efficiency; at the highest loading, further site strengthening is counterbalanced by partial blockage from excess LiOH·H_2_O phases, causing a slight decline in activity. Therefore, 0.25 Li/MgO was selected for further catalytic studies as the most effective compromise between structural properties and active site functionality.

**Table 2:** Chemical and structural properties of *x*Li/MgO catalysts^a^

Catalyst	Molar ratio (Li/Mg)	Surface area (m^2^ g^−1^)	Yield of BA (%)
0.10 Li/MgO	0.10	130	32.67
0.15 Li/MgO	0.15	88	29.41
0.20 Li/MgO	0.20	23	27.77
0.25 Li/MgO	0.25	15	40.65
0.30 Li/MgO	0.30	9	38.10

a Reaction conditions: CA (0.01 mole), CA/EtOH molar ratio (1 : 25), CA/H_2_O molar ratio (1 : 83), *x*Li/MgO (0.006 g mL^−1^), 343 K, 3 h, and 600 rpm.

#### 3.2.3. Effect of the reaction conditions

**Fig. 4:**
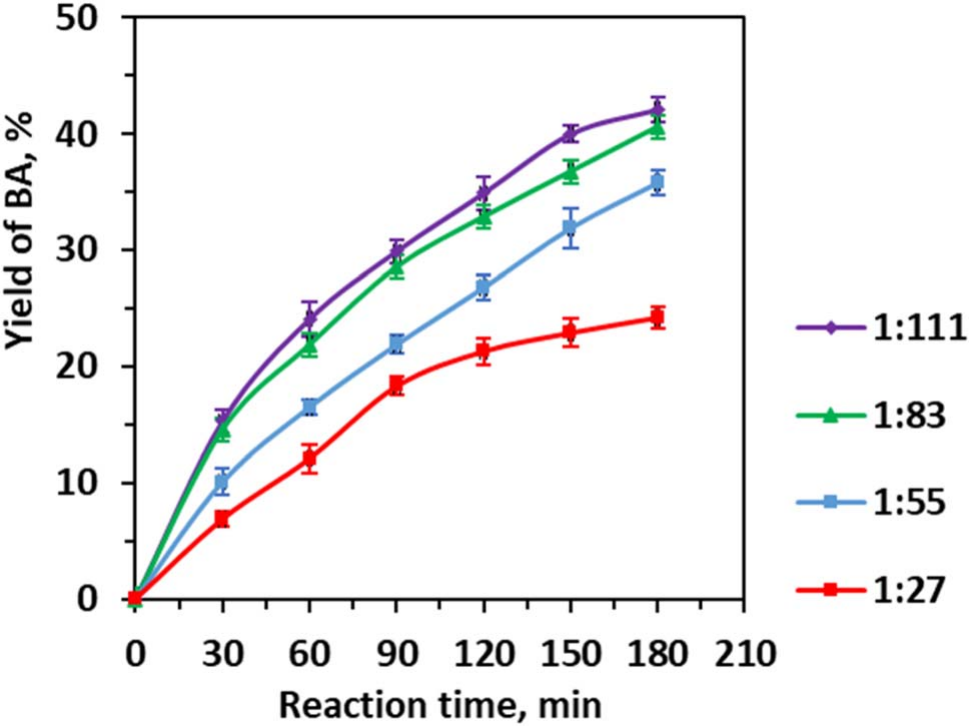
Effect of the initial molar ratio of cinnamaldehyde to water on the yield of benzaldehyde. Reaction conditions: CA (0.01 mole), CA/EtOH molar ratio (1 : 25), 0.25 Li/MgO (0.006 g mL^−1^), 343 K, 3 h, and 600 rpm.

The retro-aldol cleavage of CA proceeds within a biphasic aqueous-organic system, where CA predominantly resides in the organic phase, and water forms the continuous aqueous phase; consequently, maximizing interfacial contact and molecular dispersion is critical for catalytic efficiency. Adjusting the initial CA/H_2_O molar ratio from 1 : 27 to 1 : 111 (Fig. 4) demonstrated that increasing water content substantially enhances benzaldehyde yield by improving substrate solubility and enabling greater accessibility to active sites on the 0.25 Li/MgO catalyst. The yield increases from 24.20% at 1 : 27 to 40.65% at 1 : 83, with a plateau beyond this, reflecting the trade-off between solubility-driven reactivity and mass transfer limitations imposed by excessive aqueous phase dilution and potential phase separation. This phenomenon underscores the importance of balancing substrate concentration and solvent polarity to maintain effective reactant dispersion.

**Fig. 5:**
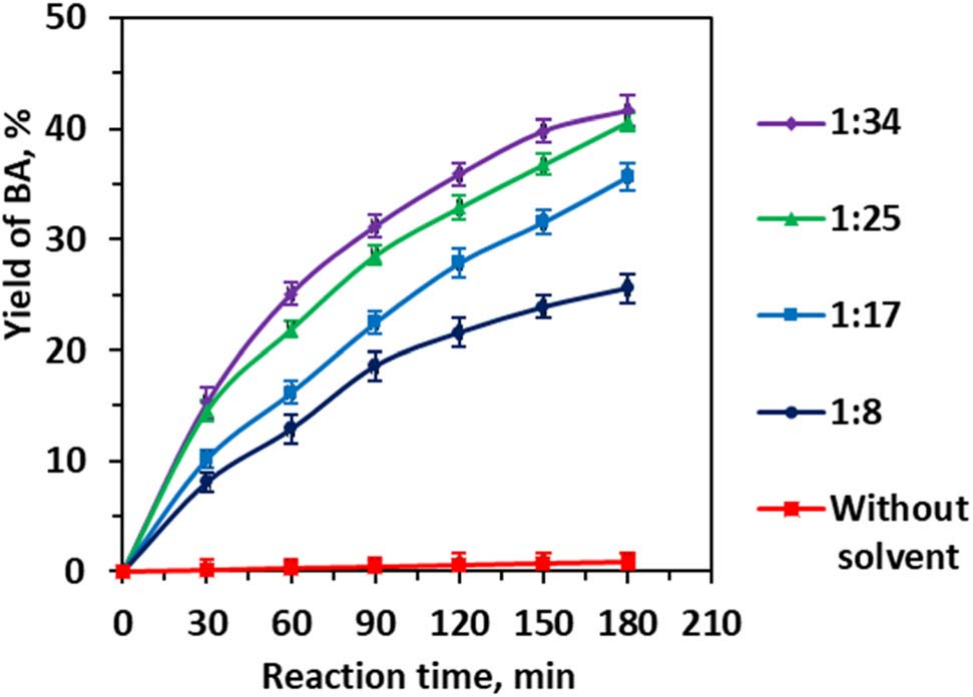
Effect of the molar ratio of cinnamaldehyde to ethanol on the yield of benzaldehyde. Reaction conditions: CA (0.01 mole), CA/H_2_O molar ratio (1 : 83), 0.25 Li/MgO (0.006 g mL^−1^), 343 K, 3 h, and 600 rpm.

To mitigate biphasic immiscibility, EtOH was introduced as a cosolvent, exploiting its amphiphilic nature to enhance substrate solubility and promote molecular-level mixing (Fig. 5). The observed yield increase from 25.60% at a CA/EtOH ratio of 1 : 8 to 40.65% at 1 : 25 illustrates EtOH's role in forming transient hydrogen bonds with CA, thereby increasing its partitioning into the aqueous phase. However, higher EtOH concentrations do not further improve yields, likely due to EtOH competing with water molecules for substrate interaction sites, which can disrupt the hydration shell critical for the retro-aldol cleavage mechanism.

**Fig. 6:**
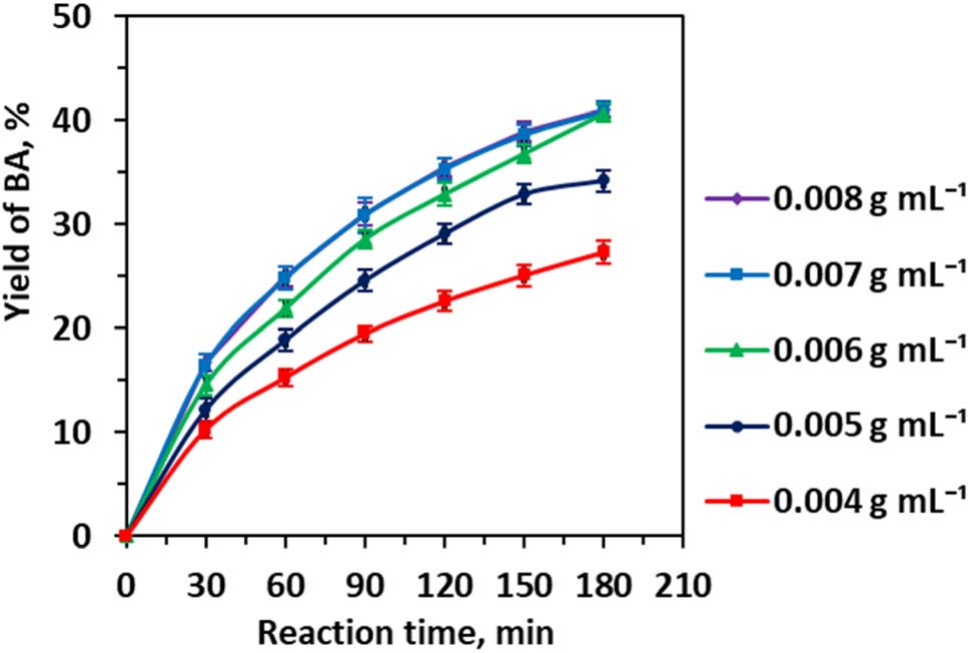
Effect of the catalyst loading on the yield of benzaldehyde. Reaction conditions: CA (0.01 mole), CA/H_2_O molar ratio (1 : 83), CA/EtOH molar ratio (1 : 25), 343 K, 3 h, and 600 rpm.

Fig. 6 shows the catalyst loading further modulates the reaction, where increasing from 0.004 to 0.006 g mL^−1^ enhances benzaldehyde yield by providing additional active sites, yet beyond this, catalyst particle agglomeration reduces the effective surface area and impairs catalyst-substrate contact, highlighting the interplay between catalyst dispersion and reaction kinetics.

**Fig. 7:**
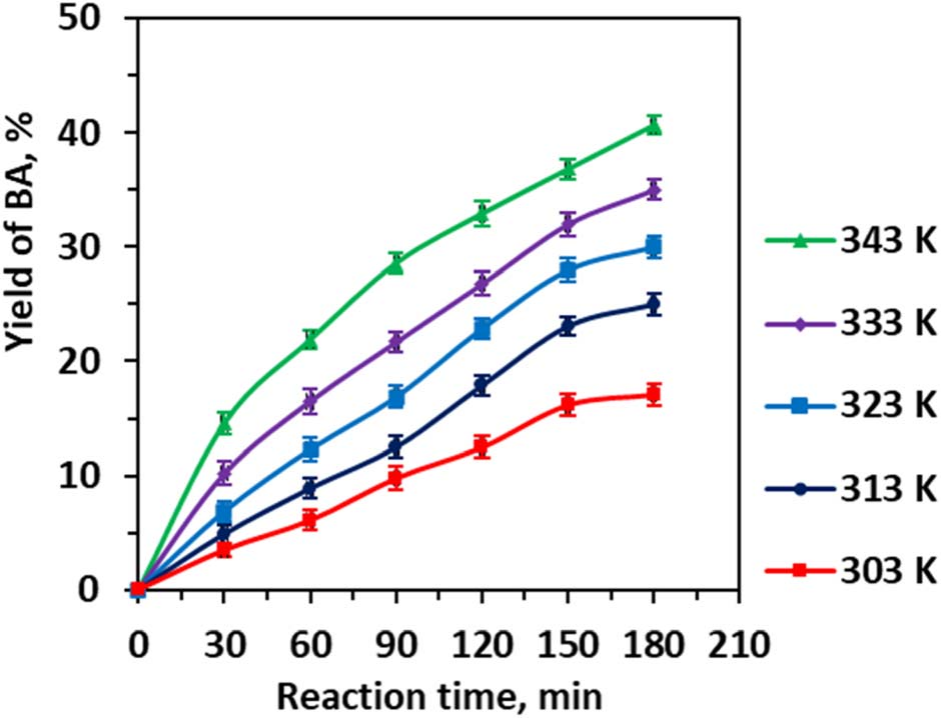
Effect of the reaction temperature on the yield of benzaldehyde. Reaction conditions: CA (0.01 mole), CA/H_2_O molar ratio (1 : 83), CA/EtOH molar ratio (1 : 25), 0.25 Li/MgO (0.006 g mL^−1^), 3 h, and 600 rpm.

Temperature exerts a profound influence (Fig. 7), with the steady yield increase from 17.12% at 303 K to 40.65% at 343 K driven by enhanced molecular collisions, accelerated reaction kinetics, and improved solvent miscibility at elevated temperatures, which collectively lower activation barriers and facilitate mass transfer across phases. Nevertheless, the upper temperature limit balances these benefits against the risk of side reactions, including benzaldehyde overoxidation or thermal degradation, preserving product selectivity and sensory qualities—a key consideration in fine chemical synthesis.

Collectively, these findings illustrate a complex, interdependent optimization landscape where solvent composition, catalyst dispersion, and thermal conditions converge to regulate solubility, phase behavior, surface accessibility, and reaction kinetics. The optimal parameters—initial CA/H_2_O molar ratio of 1 : 83, CA/EtOH molar ratio of 1 : 25, catalyst loading of 0.006 g mL^−1^, and reaction temperature of 343 K—represent a carefully balanced regime that maximizes benzaldehyde yield while maintaining catalyst efficacy and product integrity, providing valuable insights for designing efficient biphasic catalytic systems in retro-aldol and related transformations.

**Fig. 8:**
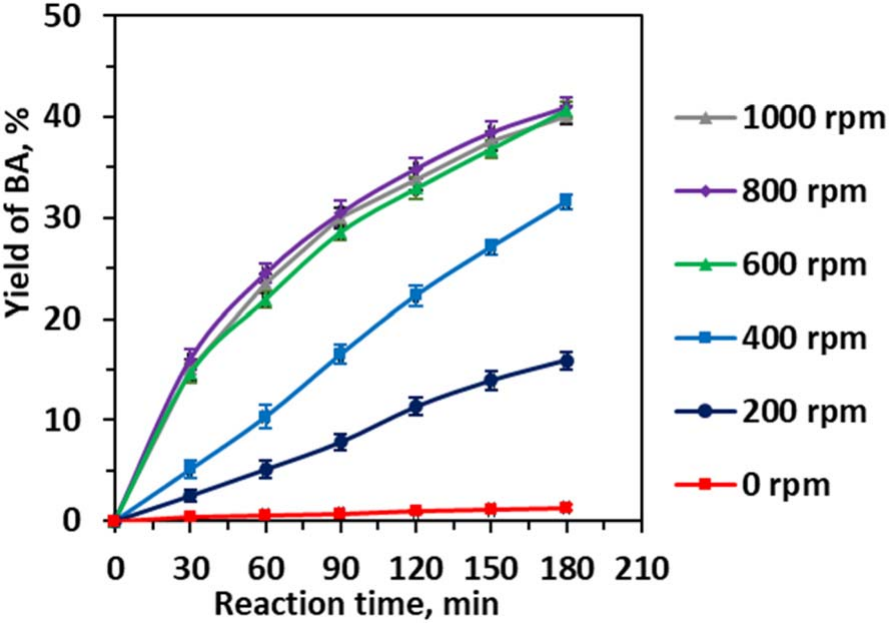
Effect of the stirring speed on the yield of benzaldehyde. Reaction conditions: CA (0.01 mole), CA/H_2_O molar ratio (1 : 83), CA/EtOH molar ratio (1 : 25), 0.25 Li/MgO (0.006 g mL^−1^), 343 K, and 3 h.

In heterogeneous catalytic reactions, effective mass transfer between reactants and catalyst surfaces is pivotal for optimizing catalytic performance.^22^ To enhance the interfacial transport of cinnamaldehyde and water across the biphasic system, stirring speed was systematically varied from 0 to 800 rpm (Fig. 8). The results reveal a pronounced dependence of benzaldehyde yield on agitation intensity. At static conditions (0 rpm), negligible product formation was observed, underscoring the critical role of mechanical mixing in overcoming mass transfer limitations. Increasing the stirring speed from 200 to 400 rpm led to a substantial rise in yield from 15.91% to 31.63%, with a further increase to 600 rpm resulting in a peak yield of 40.65%. This trend can be attributed to enhanced dispersion of the organic phase within the aqueous medium, which increases interfacial surface area and promotes efficient substrate-catalyst contact. Beyond 600 rpm, the yield plateaued, indicating that mass transfer limitations were effectively minimized and that intrinsic catalytic kinetics became rate-limiting. Notably, stirring speeds exceeding 800 rpm caused a decline in benzaldehyde yield, likely due to the formation of vortex-induced gas entrapment or reduced effective reaction volume, which can disrupt phase homogeneity and catalyst accessibility. These findings highlight the delicate balance between agitation intensity and phase behavior in biphasic catalytic systems and establish 600 rpm as the optimal stirring speed to maximize reaction efficiency without inducing detrimental hydrodynamic effects.

### 3.3. Reusability of the catalyst

**Fig. 9:**
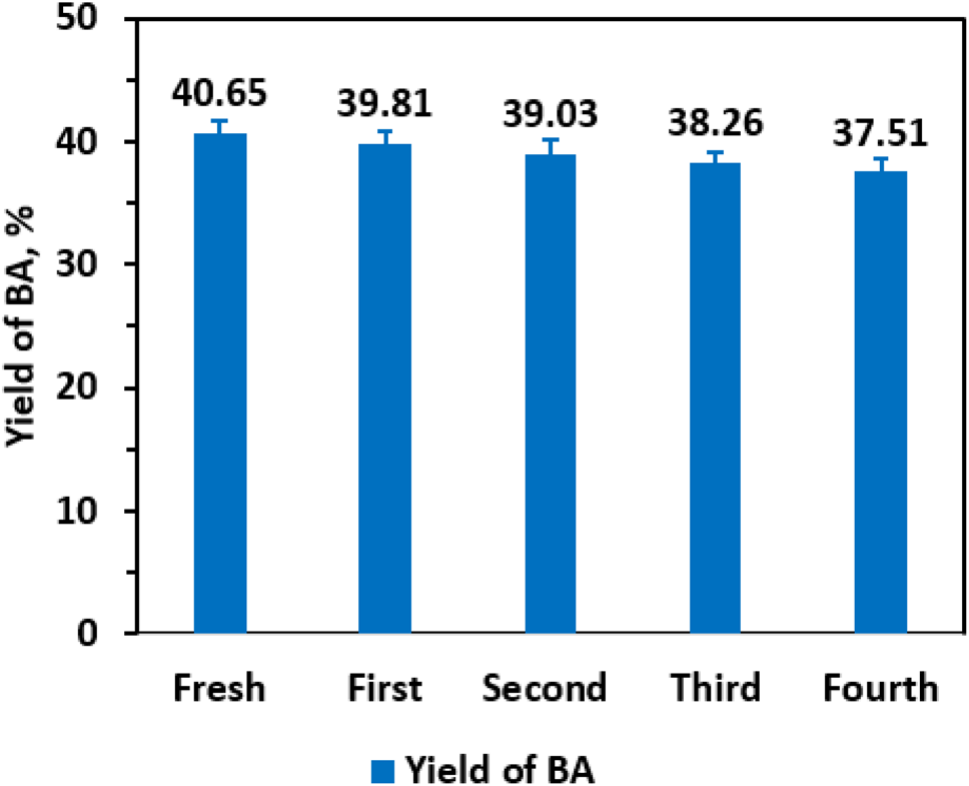
0.25 Li/MgO recyclability. Reaction conditions: CA (0.01 mole), CA/H_2_O molar ratio (1 : 83), CA/EtOH molar ratio (1 : 25), 0.25 Li/MgO (0.006 g mL^−1^), 343 K, 3 h, and 600 rpm.

Catalyst reusability and stability are critical parameters for evaluating industrial viability. To assess these, recycling experiments were conducted using the 0.25 Li/MgO catalyst under consistent reaction conditions. Upon reaction completion, the heterogeneous catalyst was efficiently recovered by centrifugation at 6000 rpm for 3 minutes at room temperature. The recovered catalyst was thoroughly washed with ethanol and deionized water to remove residual reactants and products, then dried at 393 K for 3 hours under ambient air. The dried catalyst was reused in four consecutive reaction cycles to monitor any changes in catalytic activity. As illustrated in Fig. 9, benzaldehyde yields remained essentially unchanged across all reuse cycles, with yields of 40.65%, 39.82%, 39.55%, and 39.21% for the first to fourth runs, respectively, indicating excellent catalyst stability and preservation of active sites. Furthermore, ICP-MS results showed that Li and Mg concentrations in the reaction filtrates were below the detection limit (<0.01 mg L^−1^), confirming negligible metal leaching. The constant pH value (4.36) of the reaction suspension before and after each catalytic cycle also supports the absence of metal ion release. Moreover, XRD analysis of the used catalyst revealed no detectable structural change compared with the fresh material, demonstrating excellent phase and compositional stability during reuse. This robustness is likely attributable to the strong interaction between lithium species and the MgO support, which prevents catalyst leaching and structural degradation under reaction conditions. These findings demonstrate that the 0.25 Li/MgO catalyst not only exhibits high catalytic efficiency but also maintains performance over multiple cycles, highlighting its practical potential for sustainable industrial applications where catalyst longevity and recyclability are essential for economic and environmental benefits.

### 3.4. Kinetic model

Given the large excess of water relative to cinnamaldehyde, the reverse reaction can be neglected throughout the process. The overall reaction is expressed as:1
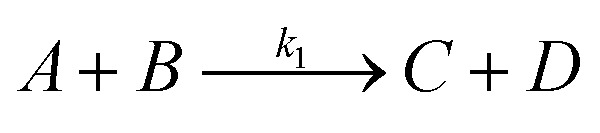
where *A*, *B*, *C*, and *D* represent cinnamaldehyde, water, benzaldehyde, and acetaldehyde, respectively, and *k*_1_ is the forward reaction rate constant. Following the pseudo-first-order kinetic model proposed by Yadav *et al.* (2013), the rate of cinnamaldehyde consumption is described by:2
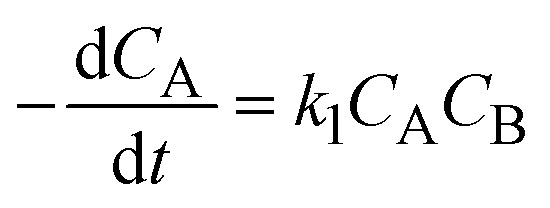
where *C*_A_ and *C*_B_ denote the molar concentrations of cinnamaldehyde and water, respectively, and *t* is the reaction time. Since water is present in significant excess, its concentration *C*_B_ remains effectively constant during the reaction. Thus, the rate expression can be simplified by defining a pseudo-first-order rate constant *K* = *k*_1_*C*_B_, yielding:3
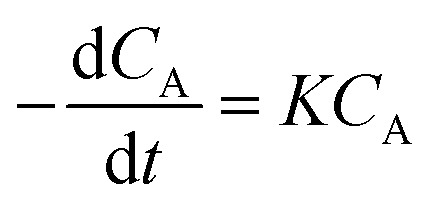


Substituting *C*_A_ = *C*_A_0__(1 − *u*_A_), where *C*_A_0__ is the initial concentration of cinnamaldehyde and *u*_A_ its conversion, eqn (3) can be rewritten as:4
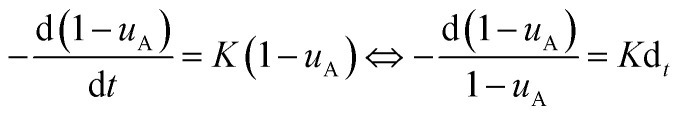


Integrate both sides of eqn (4):

5 −ln(1 − *u*_A_) = *Kt*

**Fig. 10:**
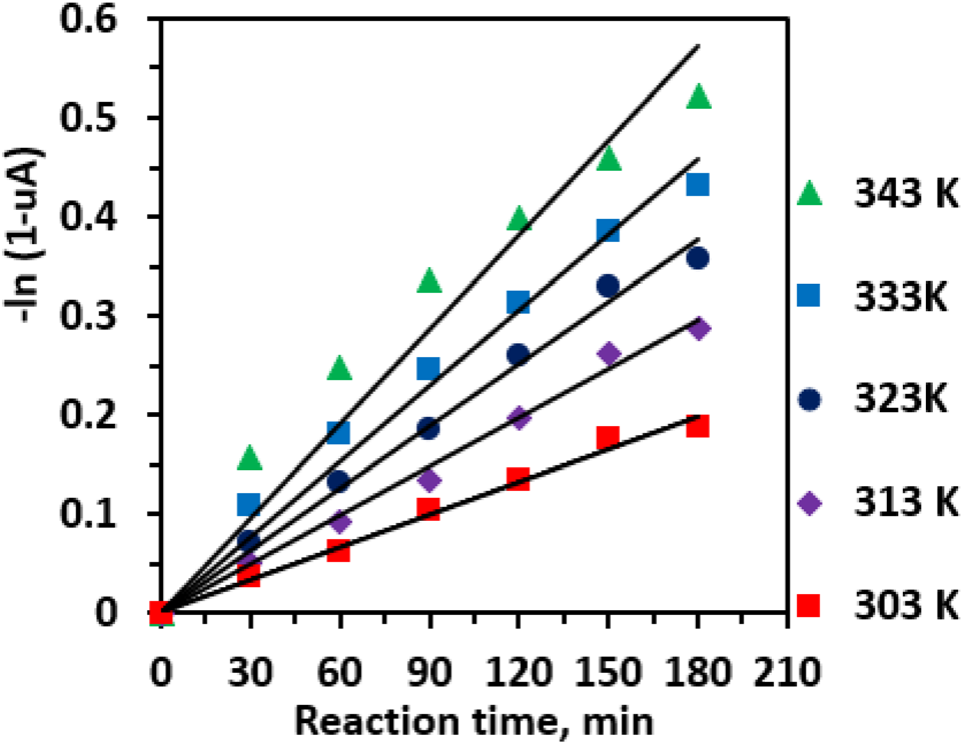
Kinetic plots at various reaction temperatures. Reaction conditions: CA (0.01 mole), CA/H_2_O molar ratio (1 : 83), CA/EtOH molar ratio (1 : 25), 0.25 Li/MgO (0.006 g mL^−1^), 3 h, and 600 rpm.

Plotting −ln(1 − *u*_A_) against reaction time *t* at various temperatures yields straight lines passing through the origin (Fig. 10), confirming the reaction's pseudo-first-order kinetics. The slopes of these lines correspond to the rate constants *K*, which increase from 0.0011 min^−1^ at 303 K to 0.0032 min^−1^ at 343 K, as summarized in Fig. 10. This temperature dependence reflects the accelerated reaction kinetics at elevated temperatures, consistent with Arrhenius behavior.

**Fig. 11:**
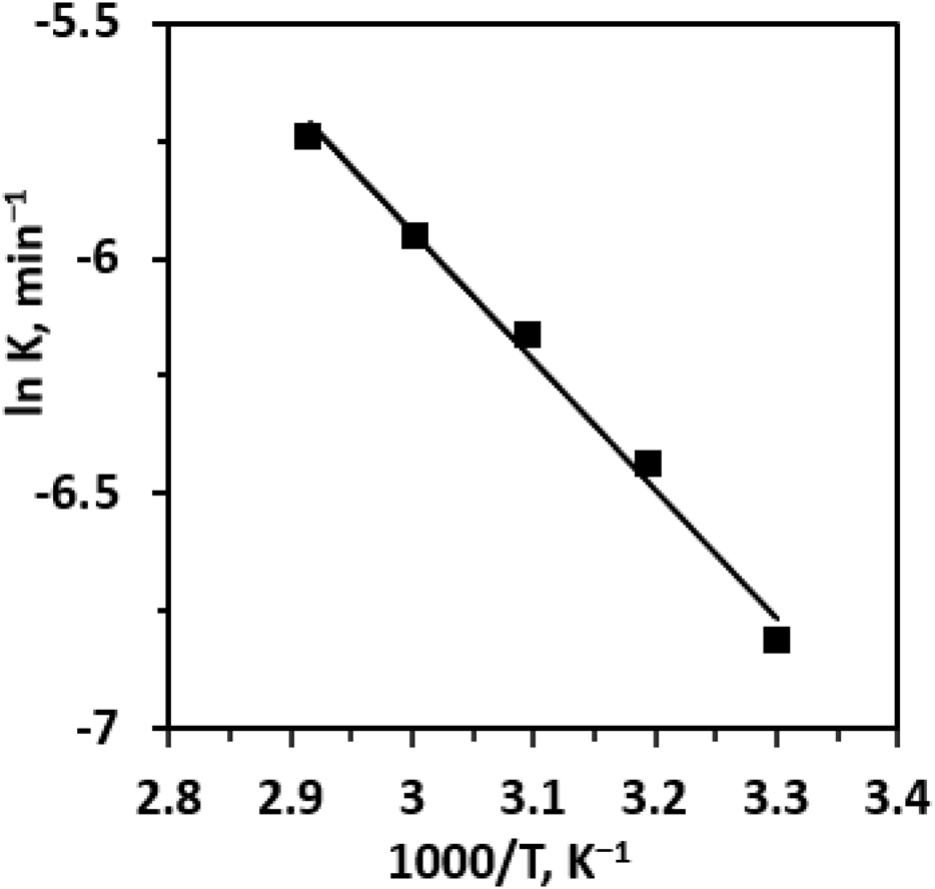
Arrhenius plot of the rate constant for the hydrolysis of cinnamaldehyde.

An Arrhenius plot of ln *K versus* 1000/*T* (Fig. 11) was constructed, yielding a straight line whose slope corresponds to −*E*_a_/*R*. From the slope, the apparent activation energy *E*_a_ was determined to be 22.73 kJ mol^−1^. This comparatively low activation energy reflects the high catalytic efficiency of the 0.25 Li/MgO catalyst.

A comparative assessment of the activation energies reported for various catalytic systems in previous studies. Table 3 clearly demonstrates that the 0.25 Li/MgO catalyst possesses the lowest activation barrier (22.73 kJ mol^−1^) for the hydrolysis of cinnamaldehyde to benzaldehyde. This value is substantially lower than that obtained with other catalysts. In addition to its kinetic advantage, the 0.25 Li/MgO catalyst enables the reaction to proceed under mild conditions (343 K, 1 atm) with a reaction time of only 3 h, achieving a benzaldehyde yield of 40.65%. Although some homogeneous systems, such as [N1111][Pro], exhibit higher yields, they often require higher catalyst loadings (up to 5 wt%) and lack recyclability. Similarly, β-CD-based systems (2-HPβ-CD/NaOH, β-CD/NaOH) demand longer reaction times (6–18 h) or higher temperatures to achieve comparable performance. In contrast, the 0.25 Li/MgO catalyst maintains a favorable balance between yield, reaction time, and operational simplicity. Moreover, the reusability of 0.25 Li/MgO over four successive cycles with negligible loss of activity highlights its superior structural and chemical stability compared with homogeneous catalysts, which are typically challenging to recover post-reaction. Taken together, these results confirm that the 0.25 Li/MgO catalyst combines the lowest activation energy, rapid conversion rate, mild reaction conditions, and excellent recyclability, underscoring its potential as a robust and sustainable catalyst for the eco-efficient production of natural benzaldehyde.

**Table 3:** The activation energy (*E*_a_) values and reaction conditions for cinnamaldehyde hydrolysis in the presence of different catalyst systems

Catalysts	*E*_a_ (kJ mol^−1^)	Yield of BA (%)	Recycle time, yield of BA change	Reaction conditions				Catalyst type	Ref.
				Catalyst loading	*T* (K)	*P* (atm)	Time (h)		
0.25 Li/MgO	22.73	40.65	4, essentially unchanged	0.006 (g mL^−1^)	343	1	3	Heterogeneous catalyst	This study
[N1111][Pro]	38.3	94	4, essentially unchanged	5 (wt%)	333	1	1	Homogeneous catalyst	15
2-HPβ-CD/NaOH	41.72	70	Without recycle	CA : 2-HPβ-CD = 1 : 1 (molar ratio), 2% NaOH (w/v)	323	1	6	Homogeneous catalyst	17
Al–Mg hydrotalcite	41.84	70	3, essentially unchanged	0.005 (g mL^−1^)	403	—	4	Heterogeneous catalyst	14
β-CD/NaOH	45.27	42	3, essentially unchanged	CA : β-CD = 1 : 1 (molar ratio), 2% NaOH (w/v)	323	1	18	Homogeneous catalyst	34
Zn-HT-glycine	80.12	75.8	3, essentially unchanged	0.005 (g mL^−1^)	403	1	4	Heterogeneous catalyst	6

### 3.5. Propose a plausible mechanism for the reaction

**Fig. 12:**
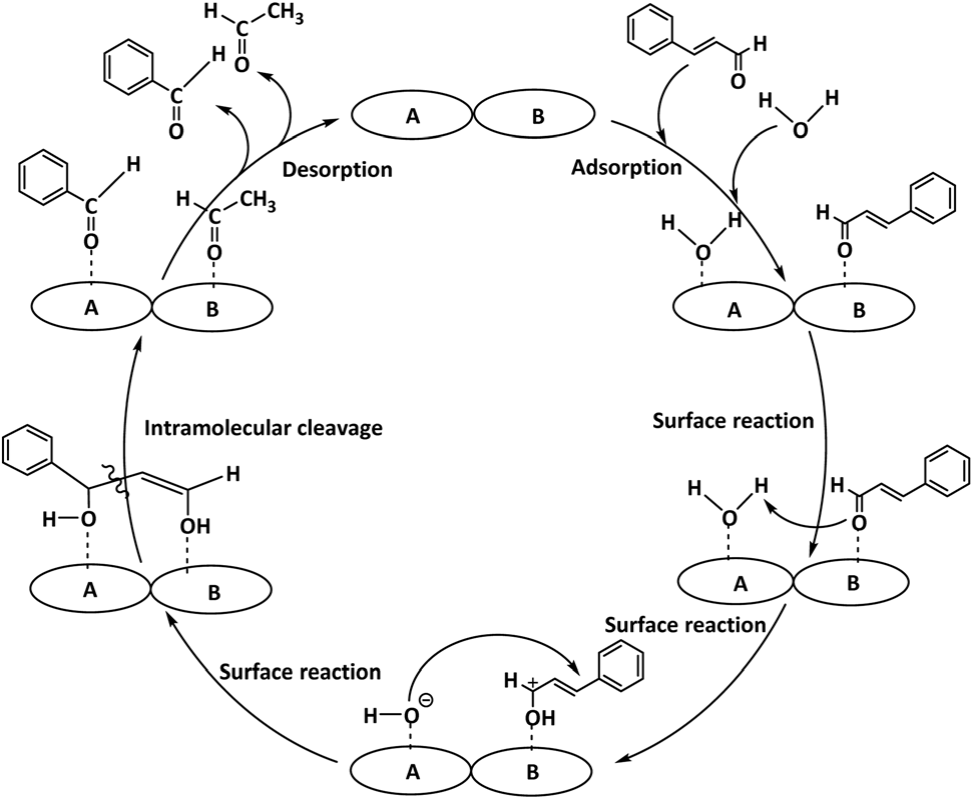
A plausible mechanism for the reaction.

The 0.25 Li/MgO catalyst exhibits bifunctional properties, possessing both acidic (A) and basic (B) active sites, which collaboratively facilitate the hydrolysis of cinnamaldehyde. Based on this dual functionality, a reaction mechanism was proposed (Fig. 12). Initially, CA molecules adsorb preferentially onto the basic sites (B), while water molecules are adsorbed onto the acidic sites (A). The CA molecule exhibits increased acidity due to the presence of strong electron-withdrawing groups, enabling it to abstract a proton from the water molecule adsorbed on the acidic sites, thereby generating a carbocation intermediate on the CA. Subsequently, the hydroxide ion—formed from the dissociation of water on the basic sites—attacks the electrophilic carbon center of the carbocation. This proton transfer between acidic and basic sites promotes a surface-mediated reaction between CA and water. The ensuing intramolecular cleavage involves the homolytic or heterolytic breaking of the C–C bond within the CA molecule, resulting in the formation of benzaldehyde on the acidic sites (A) and acetaldehyde on the basic sites (B). Finally, desorption of these products from the catalyst surface completes the catalytic cycle, regenerating the active sites for subsequent reaction cycles. This bifunctional mechanism highlights the synergistic interaction between acid and base sites on the 0.25 Li/MgO catalyst, which is essential for facilitating efficient retro-aldol cleavage under mild reaction conditions. The superior performance of Li/MgO compared with other alkali-doped or pristine MgO catalysts can be attributed to its bifunctional surface nature. The coexistence of medium-strength basic O^2−^ sites and weak acidic surface hydroxyls enables simultaneous activation of both the carbonyl and β-C–H groups in cinnamaldehyde, thus facilitating the retro-aldol cleavage pathway. This dual-site synergy is consistent with the observed low activation energy (22.73 kJ mol^−1^) and high product selectivity, highlighting the mechanistic advantage of Li incorporation into MgO.

Compared with previously reported basic catalysts such as hydrotalcites^9^ and ionic-liquid systems^10,12^ the Li/MgO catalyst exhibits a unique bifunctional surface character. The incorporation of Li^+^ ions into the MgO lattice increases the density and strength of surface O^2−^ basic sites while introducing weak acidic hydroxyls. This dual functionality enables simultaneous activation of both the carbonyl (C=O) and β-C–H bonds in cinnamaldehyde, thereby facilitating retro-aldol cleavage with a much lower activation energy (22.73 kJ mol^−1^) than that of hydrotalcite (41.8 kJ mol^−1^) or ionic-liquid catalysts (38–45 kJ mol^−1^).^9,10,13^ These mechanistic distinctions highlight the cooperative acid–base synergy on the Li/MgO surface as the key factor governing its superior catalytic efficiency under mild and green conditions.

### 3.6. Scale-up experiment

**Scheme 2:**
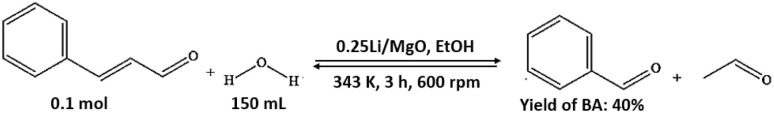
A scale-up experiment of the conversion of CA to BA catalyzed by 0.25 Li/MgO.

To assess the scalability and practical applicability of the developed catalytic system, a scale-up experiment was performed for the hydrolysis of cinnamaldehyde to benzaldehyde using the 0.25 Li/MgO catalyst and ethanol as solvent, maintaining the optimized molar ratio of reactants (Scheme 2). Notably, increasing the reaction scale by a factor of ten did not lead to any significant change in benzaldehyde yield, which remained consistently high at approximately 40%. This demonstrates that the catalytic performance and reaction efficiency are preserved upon scale-up, highlighting the robustness and reproducibility of the process. These findings underscore the strong potential of the 0.25 Li/MgO mediated hydrolysis system for industrial-scale production of benzaldehyde, offering an efficient and scalable route for sustainable synthesis of this valuable aromatic aldehyde.

## 4. Conclusions

The green synthesis of natural benzaldehyde from cinnamaldehyde was successfully achieved using Li-doped MgO catalysts prepared *via* a straightforward method. Among the catalysts tested, 0.25 Li/MgO exhibited the best performance, delivering a benzaldehyde yield of 40.65% after 3 hours under mild reaction conditions, which helps preserve the natural essence of the product. Systematic investigations of reaction parameters revealed that the hydrolysis follows pseudo-first-order kinetics with an apparent activation energy of 22.73 kJ mol^−1^ at fixed catalyst loading. Furthermore, the 0.25 Li/MgO catalyst demonstrated excellent stability and reusability over four consecutive cycles without significant loss in activity. These results highlight the catalyst's potential for industrial application in sustainable benzaldehyde production. However, despite this promising progress, the benzaldehyde yield remains moderate and warrants further optimization to enhance efficiency and meet industrial expectations. Future work will focus on catalyst design and process intensification to improve product yield and scalability.

## Author contributions

Conceptualization: N. Q. P.; methodology: N. Q. P. and H. D. T.; software: N. Q. P. and T. U. N.; validation: N. Q. P., D. C. P., T. U. N.; data curation: N. Q. P. and H. D. T.; writing original draft preparation: N. Q. P. and D. C. P.; writing – review and editing: D. V. D. , T. T. M. N. and H. D. T.; visualization: N. Q. P., D. V. D. , T. T. M. N. and H. D. T. All authors have read and agreed to the published version of the manuscript.

## Conflicts of interest

The authors declare that they have no conflict of interest.

## Supplementary Material

RA-015-D5RA07239E-s001

## Data Availability

All data obtained have been included in the manuscript and are available from the corresponding author upon reasonable request. Additional data related to this paper may be requested from the corresponding author at duc.tahong@hust.edu.vn.
