# Supplementary material for: Green and mild retro-aldol synthesis of natural benzaldehyde from cinnamaldehyde over Li-doped MgO catalysts
Source: RSC Adv. 2025 Nov 17;15(52):44680–90. doi: 10.1039/d5ra07239e (PMC12621221; doi:10.1039/d5ra07239e)
Supplement: RA-015-D5RA07239E-s001 [file RA-015-D5RA07239E-s001.pdf]

Supplementary Information (SI) for RSC Advances.
This journal is © The Royal Society of Chemistry 2025

Green and Mild Retro-Aldol Synthesis of Natural Benzaldehyde from Cinnamaldehyde over Li-Doped MgO Catalysts

Ngoc Quang Phan[a], Duc Chinh Pham[a], Thi Uyen Nguyen[a], Dang Van Do[b], Thu Thi Minh Nguyen[b], and Hong Duc Ta[*a]

[a] *School of Chemistry and Life Science, Hanoi University of Science and Technology, No. 1 Dai Co Viet, 100000, Hanoi, Vietnam.*

[b]*Faculty of Chemistry, VNU University of Science Ha Noi, No. 19 Le Thanh Tong, Ha Noi, Viet Nam*

Email: duc.tahong@hust.edu.vn

1. List of Figures and Tables

Figure S1. X-ray diffraction patterns of MgO and xLi/MgO.
Figure S 2. XRD pattern of 0.25Li/MgO catalyst before and after the reaction.

Table S1. Chemical and structural properties of MgO and xLi/MgO catalysts.
Table S2. pH of the reaction solution before and after the catalyst distribution.
Table S3. Surface acid and base site properties of MgO and x-Li/MgO samples.

1.1. XRD data

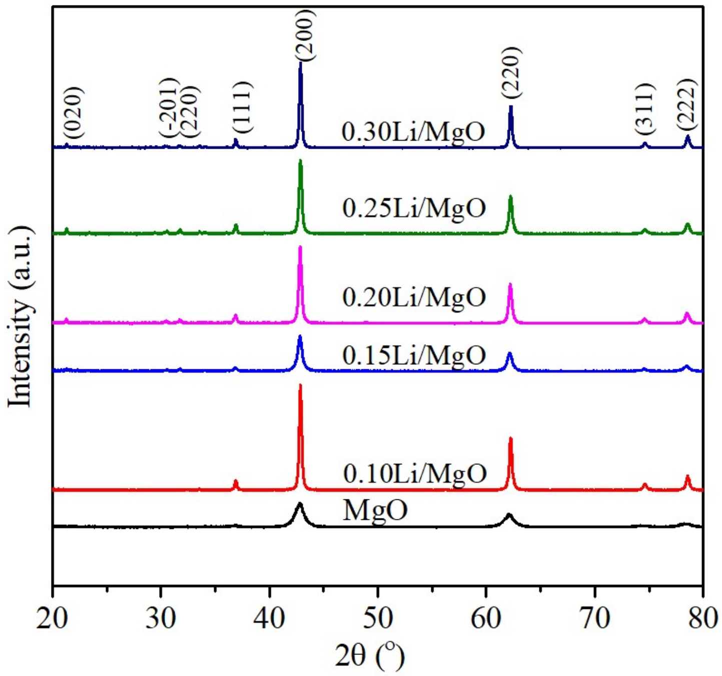

Figure S1. X-ray diffraction patterns of MgO and xLi/MgO.

Table S1. Chemical and structural properties of MgO and xLi/MgO catalysts.

Catalyst	Crystallite sizes (nm)
MgO	8.38
0.10Li/MgO	30.98
0.15Li/MgO	15.76
0.20Li/MgO	25.19
0.25Li/MgO	25.54
0.30Li/MgO	31.53

1.2. Checking the metal leakage

Table S2. pH of the reaction solution before and after the catalyst distribution.

Recycle experiment	Fresh		First		Second		Third		Fourth	
	Before	After	Before	After	Before	After	Before	After	Before	After
pH value	4.36	4.36	4.36	4.36	4.36	4.36	4.36	4.36	4.36	4.36

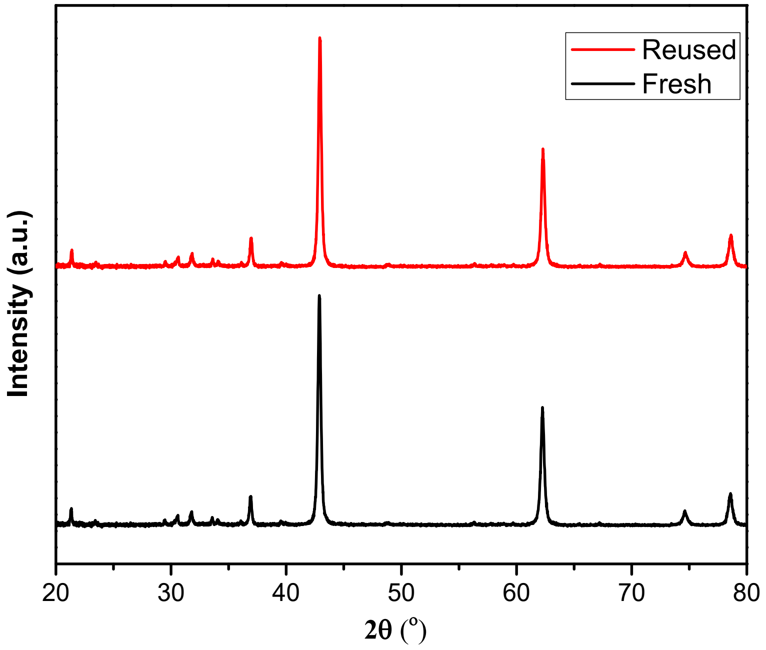

Figure S 2. XRD pattern of 0.25Li/MgO catalyst before and after the reaction.

1.3. Numerical integration of CO_2 – and NH_3-TPD curves

Table S3. Surface acid and base site properties of MgO and x-Li/MgO samples.

Catalyst	Total basic sites (mmol g^{-1})	Total acidic sites (mmol g^{-1})	Remarks
MgO	0.42	0.15	Weak–moderate basicity; few acid sites
0.10Li/MgO	0.50	0.18	Increased basic site density
0.25Li/MgO	0.57	0.21	Well-balanced bifunctional acid–base sites
0.50Li/MgO	0.53	0.19	Slight decrease due to Li over-loading
